# Early Detection of Cardiac Damage by Two-Dimensional Speckle Tracking Echocardiography After Thoracic Radiation Therapy: Study Protocol for a Prospective Cohort Study

**DOI:** 10.3389/fcvm.2021.735265

**Published:** 2022-01-26

**Authors:** Dan Zhu, Tingcui Li, Hongqing Zhuang, Ming Cui

**Affiliations:** ^1^Department of Cardiology, Peking University Third Hospital, Beijing, China; ^2^Department of Radiation Oncology, Peking University Third Hospital, Beijing, China

**Keywords:** radiotherapy, radiotherapy-induced heart disease, two-dimensional speckle tracking echocardiography, biomarkers, cardiotoxicity

## Abstract

**Background:**

As one of the important treatment methods for cancer patients, radiotherapy may lead to incidental irradiation of the heart, resulting in radiotherapy-induced heart disease (RIHD) arising many years after radiotherapy. While, there are few studies on early subclinical cardiac damage, which may be essential for the protection of late RIHD. To detect and predict RIHD and early subclinical cardiac damage induced by thoracic radiation therapy, based on two-dimensional speckle tracking echocardiography (2D STE) combined with multiple circulating biomarkers and accurate heart dosimetry.

**Methods and Analysis:**

This is a monocentric prospective cohort study in which 104 patients treated for malignant tumors and with cardiac radiation exposure will be included. All participants will be followed for 12 months after radiotherapy. Echocardiography, 2D STE, and blood samples will be underwent at 5-time points (baseline; after completion of RT; 2, 6, and 12 months after RT). Left ventricular ejection fraction (LVEF); global longitudinal, radial, and circumferential strain; diastolic function parameters; creatine kinase (CK); creatine kinase isoenzyme (CK-MB); cardiac troponin T (cTnT); N-terminal pro-B-type natriuretic peptide (NT-proBNP) and hypersensitive C-reactive protein (hs-CRP) will be measured at baseline and every follow-up time. The incidence of major adverse cardiovascular events will be recorded.

**Discussion:**

This study details the protocol and presents the primary limits and advantages of this single-center project. The inclusion of patients began in 2021, and the results are expected to be published in 2023. This study will be allowed to enhance knowledge on detection and prediction of early subclinical cardiac dysfunction induced by thoracic radiation therapy, based on two-dimensional speckle tracking echocardiography (2D STE) combined with circulating biomarkers and accurate heart dosimetry. Furthermore, we will evaluate risk factors of subtle cardiac damage and identify high-risk groups for early heart damage.

**Clinical Trial Registration:**

ClinicalTrials.gov, identifier: NCT04443400.

## Introduction

Cancer is expected to rank as the most critical barrier to increasing life expectancy and the leading cause of death in the world in the twenty-first century. Data from global cancer statistics indicate that there will be about 18.1 million new cancer patients all over the world in 2018 (1, 2). According to statistics, one half of the cancer patients would undergo radiation therapy (RT) as neoadjuvant or adjuvant treatment (3). As an essential treatment modality of comprehensive treatment for cancer patients, radiotherapy can significantly improve the cure rate and survival rate of cancer patients (4). However, RT used to treat carcinomas, especially thoracic cancer, such as Hodgkin lymphoma, lung, breast cancer, etc, carries a high risk of developing cardiovascular side effects.

Heart is inevitably affected during thoracic radiation therapy, resulting in radiotherapy-induced heart damage. Thoracic radiation therapy is associated with long-term increased risk of coronary artery disease, heart failure, myocardial infarction, valvular disease, arrhythmias, pericarditis, and major adverse cardiovascular events many years after RT (5, 6). A meta-analysis of more than 42,000 breast cancer patients in 78 randomized controlled trials showed heart disease mortality increased in patients received RT (rate ratio 1.27, 2p = 0.0001) (7). Several studies confirmed the association between radiotherapy and the increased risk of heart disease, showed the relative risks within the range of 1.18–3.5 (8–11). A retrospective study of 2168 women undergoing radiotherapy for breast cancer showed that the rates of major coronary events increased linearly with the mean dose to the heart by 7.4% per gray (95% confidence interval, 2.9 to 14.5; *P* < 0.001), with no minimum threshold for risk (10). Moreover, a retrospective single-institution study of 415 patients with Hodgkin lymphoma showed that the incidence of coronary artery disease 20 years later to be 10.4% after a median of 9 years after RT treatment (12). In general, compared with non-irradiated patients, patients undergoing thoracic radiotherapy have a 2% higher absolute risk of cardiac morbidity at 5 years and a 23% increased absolute risk after 20 years (4). Radiotherapy-induced heart disease (RIHD) can involve any structure of the heart and has become one of the major complications threatening the survival of post-RT cancer patients (4, 12–14). Heart complications may partially offset the positive effect of radiation therapy on cardiac radiation exposure patients.

Long before the onset of clinically cardiac events occurring, subclinical cardiac changes may occur during the process, after completion, over weeks, months, or first years after RT, that can be detected based on functional dysfunction (15–17). Early detection of RIHD may have important clinical significance for cancer patients, especially young cancer patients. Furthermore, it may be essential for the prediction and protection of late RIHD.

As an essential tool for the assessment of cardiac structure and function, echocardiography has been widely used. However, left ventricular ejection fraction (LVEF) is a relatively insensitive index for detecting subclinical cardiac damage at an early stage (15, 17). It is mainly because no significant change in LVEF occurs until a critical amount of myocardial damage took place and after compensatory mechanisms are exhausted. Recently, two-dimensional speckle-tracking echocardiography (2D STE) has been used for detecting and quantifying subtle cardiac damage induced by RT. Previous studies have shown that when the radiation reaches a specific dose, global longitudinal, circumferential strain, radial strain, and strain rate are substantially decreased, which can be detected by 2D STE before the decrease of LVEF and the appearance of clinical symptoms (16–18). The most recent study included 40 women treated for left breast cancer showed that global longitudinal strain (GLS) significantly decreased during the first year following RT: a decrease in strain was observed at all post-RT time points (18.9 ± 2 at 6 weeks, 18.9 ± 3 at 12 months vs. 20.6 ± 2% before RT, *p* < 0.01) (17). Based on this clinical state, it appeared that early subclinical cardiac dysfunction of RT could be measured by strain, which is consistent with recent recommendations in the evaluation of cardiovascular complications after radiotherapy (19).

Traditional circulating biomarkers, such as creatine kinase (CK), creatine kinase isoenzyme (CK-MB), cardiac troponin T (cTnT), and cardiac troponin I (cTnI) may play an essential role in the detection of acute and late RIHD (20–22). However, early subclinical cardiac damage does not necessarily have an increase in these cardiac biomarkers. Fortunately, many serum biomarkers (e.g., hypersensitive C-reactive protein (hs-CRP) and N-terminal pro-B-type natriuretic peptide (NT-proBNP) have been shown to be potential biomarkers for cardiac damage after RT (22–24). Despite the variability of outcomes, specific serum biomarkers may represent a promising tool for the prediction of early myocardial dysfunction, leading to a more appropriate stratification of those patients who demand closer monitoring. Nevertheless, further studies with more specific biomarkers are certainly needed to empower this association so that patients receive the greatest benefit.

A large number of studies have shown that many chemotherapy drugs can lead to cardiac toxicity (25, 26). Patients with chemotherapy exposure before radiation therapy may be more likely to present radiation-induced heart damage. The history of chemotherapy may be an essential risk factor for radiation-induced heart damage. However, to our knowledge, there is no prospective study on the occurrence of early radiation-induced heart damage in patients with or without a history of chemotherapy. Therefore, this study aims to assess the value of 2D STE combined with the assessment of multiple circulating biomarkers and accurate heart dosimetry in the detection and prediction of early subclinical cardiac damage induced by thoracic radiation therapy. Risk factors and high-risk groups of subtle cardiac damage and major adverse cardiovascular events (MACE) during the 12 months follow-up will also be evaluated.

## Methods And Analysis

### Study Design

It is a monocenter, prospective cohort study that will include patients who will receive RT. All patients will be followed 12 months after RT. 2D STE parameters and circulating biomarkers will be collected. MACEs were defined as a diagnosis of unstable angina [*International Classification of Diseases, 10th Revision* (ICD-10) codes I20], new arrhythmia (ICD-10 codes I44–I49), acute myocardial infarction (ICD-10 codes I21), heart failure (ICD-10 codes I50), valvular heart disease (ICD-10 codes I08 and I39), acute pericarditis (ICD-10 codes I30), and cardiac death (diagnosis by clinician) in this study. The determination of maces events is based on the patient's clinical symptoms, the results of cardiac damage markers, electrocardiogram (ECG), echocardiography and ambulatory ECG (27–32).

### Study Population and Groups

One hundred and four patients with thoracic radiotherapy will be included in Peking University Third Hospital from June 21, 2020 to December 31, 2021. Participants will be recruited based on the eligibility criteria presented in Table 1. All of the patients will be included at the baseline before RT and followed for 12 months after RT. Written informed consent will be obtained from all recruited patients. This study was approved by the Ethics Committee of the Peking University Third Hospital and completed under its supervision. Informed consent was signed by all study participants. Our study was in accordance with all the ethical requirements and followed the reporting guideline for case series. Patients or the public were not involved in the design, or conduct, or reporting, or dissemination plans of our research.

**Table 1:** Inclusion and exclusion criteria.

**Inclusion criteria**	**Exclusion criteria**
With malignant tumors	Satisfactory echocardiographic images could not be obtained
Will receive radiotherapy	Moderate or severe valvular disease
With cardiac exposure during the radiotherapy process	Cardiomyopathy
Could receive regular follow-up for 12 months	Congenital heart disease
Written informed consent	Refractory hypertension
	Coronary artery disease^*^
	Heart failure
	Arrhythmia requiring intervention
	Pericarditis
	Acute myocarditis
	Participating in other clinical studies of drug intervention
	Severe liver and kidney dysfunction
	Autoimmune disease
	Pulmonary hypertension

*Coronary artery disease: at least 50% stenosis based on previous coronary angiography or coronary CT angiography, or with a history of percutaneous coronary stent implantation*.

* *With LVEF <50%, unstable angina, or acute myocardial infarction within 3 months*.

Oncologist and cardiologist will present the study to the eligible patients, explaining the study, offering participation, and requesting written informed consent. All patients will be informed that they have no obligation to participate, and they can stop participating at any time and without any consequences for therapeutic schedule and medical follow up.

### Follow-Up Strategy

Before radiotherapy, echocardiography, 2D STE, CK, CK-MB, cTnT, NT-proBNP, ECG, hs-CRP, alanine aminotransferase (ALT), aspartate aminotransferase (AST) and creatinine will be detected, and eligible patients will be enrolled. All participants will be followed up after completion of RT (post-RT), 2 months after RT (2-months post-PT), 6 months after RT (6-months post-PT), and 12 months after RT (12-months post-PT). During subsequent follow-up, echocardiography, 2D STE, CK, CK-MB, cTnT, NT-proBNP, ECG, and hs-CRP will be collected at every follow-up time.

### Data Collection

A precise description of cancer, treatments for cancer, and information about the main risk factors of a cardiac event is collected at inclusion; details will be shown in Table 2. Echocardiography, 2D STE, ECG, and circulating biomarker measurements are detailed in Table 3.

**Table 2:** Baseline characteristics.

**Characteristic**		**Value**
Age		
Hight		
Weight		
Body mass index (BMI)		
Systolic blood pressure (SBP)		
Diastolic blood pressure (DBP)		
Smoking status		
Hypertension		
Diabetes		
Hyperlipidemia		
Arrhythmia		
Cancer	Type
	Size
	Chemotherapy history
	Mean heart dose

**Table 3:** Parameters of echocardiography, 2D STE, ECG and circulating biomarkers.

**Parameters**
**Echocardiography: commercially available ultrasonography systems**
LVEF measured by the modified Simpson or the modified Quinones method
LV mass index calculated by the Devereux formula
The degree of aortic and mitral regurgitation classified according to the guidelines of the American Society of Echocardiography
E/A wave ratio
E/e' ratio
Left atrium volume index calculated using the area-length method
Systolic pulmonary arterial pressure
**Two-dimensional speckle-tracking echocardiography: TomTec (TomTec Imaging Systems GmbH, Unterschleissheim, Germany)**
Global longitudinal strain (GLS)
Global radial strain (GRS)
Global circumferential strain (GCS)
S'
**ECG**
Heart rate
rhythm
P-R intervals
QRS wave
QTC
S-T segment changes
**Circulating biomarkers**
CK
CK-MB
cTnT
NT-proBNP
HsCRP

*2D STE, two-dimensional speckle tracking echocardiography; ECG, electrocardiogram; LVEF, left ventricular ejection fraction; LV, left ventricular; CK, creatine kinase; CKMB, creatine kinase isoenzyme; cTnT, cardiac troponin T; NT-proBNP, N-terminal pro-B-type natriuretic peptide*.

### Radiation Therapy

All of the patients will be treated with stereotactic body radiotherapy (SBRT). Before radiotherapy, the chest CT examination will be carried out to locate the target area. According to the location of CT, the treatment target area is gross tumor volume and planning target volume is appropriately expanded on the basis of the anterior target. The treatment plan was made by multiplan 4.6 planning system. After the plan was completed, the plan was verified first, and then the stereotactic radiotherapy robot was used. In the treatment, we use the Xsight-Spinal tracking system, synchronous respiratory tracking mode, or the six-dimensional bed system to control the patient's position and focus perfectly, so as to achieve the effect of precise radiotherapy. According to the treatment plan, the minimum, average and maximum doses of the heart and the V5, V10, V20 and V30 of the heart are counted. To evaluate the effect of radiotherapy dose on heart more accurately, we converted all maximum and mean cardiac doses to the biologically effective doses (BED), assuming an α/β ratio of 3 for the heart, with conversion to an equivalent dose in 2-Gy fractions (EQD2) using the linear quadratic model (33–35). The BED was calculated using the equation nd[1+d/(α/β)], the EQD2 was calculated from the dose-volume histograms as nd[(d+α/β) ÷ (2+α/β)], n is a number of fractions, d is dose per fraction, and α/β was 3 Gy (36). According to the RTOG 0236 report, the average dose of heart is limited to less than or equal to 30Gy (37). Planning and contouring of the RT and the affected area and dose of heart will be recorded.

### Study Endpoints

#### Primary Endpoint

The primary endpoint is an at least 10% decrease in the global longitudinal, radial, and circumferential strain or strain rate, determined using cardiac 2D STE, compared with the baseline, which can be considered clinically pertinent based on those observed in the previous studies (38).

#### Secondary Endpoints

CK, CKMB, cTnT and NT proBNP were within the range of normal reference values at baseline, but exceeded the upper limit of normal reference values at follow-up.

ECG indicates new arrhythmia.

Patients had symptoms and signs of heart failure with LVEF decreased by at least 5% and less than 55%, or LVEF decreased by at least 10% and less than 55% without symptoms and signs, or LVEF <50%, measured by echocardiography (38).

### Data Management

Study data will be managed by two study investigators using a predesigned casereport form (Microsoft Office Excel and Word) with double data-entry. Data checks will be regularly performed to ensure data quality. The number of eligible, included and excluded of patients will be recorded. Withdrawals will also record as detailed as possible.

### Statistical Analysis

#### Sample Size Calculation

The sample size was based on a statistical power of 80%, an alpha-risk of 5%, the definition of the primary endpoint (a decrease in the global longitudinal, radial, circumferential strain or strain rate of at least 10%). The baseline value was derived from the literature: mean global longitudinal strain before RT = the post-RT value was derived from the literature: mean global longitudinal strain after RT in patients without a history of chemotherapy = −17.5 ± 1.7%, mean global longitudinal strain after RT in patients with a history of chemotherapy = −16.1 ± 1.9% (39). Taking into account a 20% patients lost, the inclusion of 104 patients is necessary.

#### Statistics

Continuous variables will be expressed as means ± standard deviation or median (interquartile range) where appropriate. Categorical variables will be expressed as counts (percentages). Changes regarding early subclinical cardiac damage and biomarkers between the baseline and the follow-up in two groups will be tested with repeated measures analysis of variance. For within-group comparisons, changes in the GLS, GCS, GRS, S', LVEF, and circulating biomarkers between the baseline and the follow-up time points will be analyzed using paired t-test for parametric variables, and Wilcoxon signed-rank test for non-parametric variables. Chi-square test will be used to compare the occurrence and progression of early subclinical cardiac damage and changes in circulating biomarkers between the two groups. Mixed regression models will be used to investigate the correlation between RIHD and age, blood pressure, BMI, smoking status, hypertension, hypercholesterolemia and heart dose. All statistical analyses will be performed using SPSS 24.0 statistical software, and a *P* < 0.05 will be considered significant.

## Discussion

This population-based cohort study aims to estimate the value of 2D STE combined with multiple circulating biomarkers in the detection and prediction of early subclinical cardiac damage induced by thoracic radiation therapy in patients with SBRT. Furthermore, we want to find the risk factors of subtle cardiac damage and to determine the prevalence of MACE within 12 months post-RT.

The study has far-reaching clinical significance. Firstly, the subclinical heart damage reduced by radiotherapy can be identified early by combining 2D STE, echocardiography and a variety of circulating biomarkers. Secondly, the risk factors of RIHD were further identified by prospective cohort study, so as to achieve the early identification of high-risk groups before radiotherapy in the future. Moreover, after detection of RIHD, we will further explore the occurrence mechanism of early RIHD, and explore the prevention and treatment measures through randomized controlled trials, in order to achieve early intervention before the occurrence of clinical symptoms, reduce the incidence of RIHD, alleviate a patient's suffering, improve the quality of patients' life and reduce the economic burden on patients and society. Meanwhile, this study will also provide reference clinical research data for the formulation of guidelines and the detection and protection of RIHD.

Meanwhile, there are some limitations to this study. First, the sample size was small,which might represent a beta error. Second, as the follow-up in this study was limited to 12 months, subclinical cardiac changes may not produce during this period. A larger study with a longer duration of follow-up would be required to validate our current observations and to determine its impact on long-term adverse outcomes. Although these hindrances should be acknowledged, we believe the findings from this study will provide important data, which will help focus on “high-risk” patients for longer follow-ups and take up protective measures.

This study will allow evaluating the advantages of 2D STE combined with circulating biomarkers in early detection and prediction of RIHD and validate risk factors and high-risk groups of subclinical cardiac damage, which will be important for proposing primary and secondary preventive measures and improving patient care and quality of life.

To summarize, this study will elucidate concerns and problems related to RIHD and risk foctors.The findings will form a basis for developing early detection method for subclinical cardiac damage and interventions to alleviate RIHD in people who will receive thoracic radiotherapy in the shaort and long term.

## Ethics Statement

Ethical approval has been obtained for the study procedures by the Ethics Committee of Peking University Third Hospital, China (IRB00006761-M2020074). Any protocol amendments will be submitted to the Ethics Committee of Peking University Third Hospital for ethical approval. Written informed consent will be collected from all participants before entering this study.

## Author Contributions

MC: conceptualization, supervision, and writing—review and editing. HZ and TL: data curation and formal analysis. DZ and TL: writing—original draft. All authors have read and agreed to the published version of the manuscript.

## Funding

This study is funded by Peking University Third Hospital (BYSYLXHG2020003). The Peking University Third Hospital has no direct role in the design, conduct, or analysis of this study.

## Conflict of Interest

The authors declare that the research was conducted in the absence of any commercial or financial relationships that could be construed as a potential conflict of interest.

## Publisher's Note

All claims expressed in this article are solely those of the authors and do not necessarily represent those of their affiliated organizations, or those of the publisher, the editors and the reviewers. Any product that may be evaluated in this article, or claim that may be made by its manufacturer, is not guaranteed or endorsed by the publisher.
